# Complex Alterations of Fatty Acid Metabolism and Phospholipidome Uncovered in Isolated Colon Cancer Epithelial Cells

**DOI:** 10.3390/ijms22136650

**Published:** 2021-06-22

**Authors:** Jiřina Hofmanová, Josef Slavík, Miroslav Ciganek, Petra Ovesná, Zuzana Tylichová, Martina Karasová, Ondřej Zapletal, Nicol Straková, Jiřina Procházková, Jan Bouchal, Zdeněk Kolář, Jiří Ehrmann, Monika Levková, Zlatka Hušková, Pavel Skalický, Alois Kozubík, Miroslav Machala, Jan Vondráček

**Affiliations:** 1 Department of Cytokinetics, Institute of Biophysics of the Czech Academy of Sciences, 612 65 Brno, Czech Republic; hofmanova@ibp.cz (J.H.); ovesna@iba.muni.cz (P.O.); tylichova@ibp.cz (Z.T.); karasova@ibp.cz (M.K.); ondzap@cktch.cz (O.Z.); Nicol.Strakova@seznam.cz (N.S.); prochazkova@ibp.cz (J.P.); kozubik@ibp.cz (A.K.); 2 Department of Experimental Biology, Faculty of Science, Masaryk University, 625 00 Brno, Czech Republic; 3 Department of Pharmacology and Toxicology, Veterinary Research Institute, 621 00 Brno, Czech Republic; slavik@vri.cz (J.S.); ciganek@vri.cz (M.C.); 4 Institute of Biostatistics and Analyses, Faculty of Medicine, Masaryk University, 602 00 Brno, Czech Republic; 5 Department of Clinical and Molecular Pathology, Institute of Molecular and Translational Medicine, Faculty of Medicine, Palacký University and University Hospital, 779 00 Olomouc, Czech Republic; jan.bouchal@upol.cz (J.B.); kolarz@tunw.upol.cz (Z.K.); monika.levkova@upol.cz (M.L.); zlata.huskova@upol.cz (Z.H.); 6 Department of Surgery, University Hospital, 779 00 Olomouc, Czech Republic; jiri.ehrmann2@fnol.cz (J.E.); pavel.skalicky@fnol.cz (P.S.)

**Keywords:** phospholipids, lysophospholipids, fatty acid synthesis, desaturation, colorectal carcinoma, EpCAM, epithelial cells, lipidomics

## Abstract

The development of colon cancer, one of the most common malignancies, is accompanied with numerous lipid alterations. However, analyses of whole tumor samples may not always provide an accurate description of specific changes occurring directly in tumor epithelial cells. Here, we analyzed in detail the phospholipid (PL), lysophospholipid (lysoPL), and fatty acid (FA) profiles of purified EpCAM^+^ cells, isolated from tumor and adjacent non-tumor tissues of colon cancer patients. We found that a number of FAs increased significantly in isolated tumor cells, which also included a number of long polyunsaturated FAs. Higher levels of FAs were associated with increased expression of FA synthesis genes, as well as with altered expression of enzymes involved in FA elongation and desaturation, including particularly fatty acid synthase, stearoyl-CoA desaturase, fatty acid desaturase 2 and ELOVL5 fatty acid elongase 5 We identified significant changes in ratios of specific lysoPLs and corresponding PLs. A number of lysophosphatidylcholine and lysophosphatidylethanolamine species, containing long-chain and very-long chain FAs, often with high numbers of double bonds, were significantly upregulated in tumor cells. Increased *de novo* synthesis of very long-chain FAs, or, altered uptake or incorporation of these FAs into specific lysoPLs in tumor cells, may thus contribute to reprogramming of cellular phospholipidome and membrane alterations observed in colon cancer.

## 1. Introduction

The high incidence and mortality of colon cancer, in particular in high-income countries [1], requires improved tools for its early diagnosis and treatment. Over the recent years, it became evident that genetic events in cancer also lead to aberrant cellular metabolism, which enable cancer cells to utilize alternate energy resources [2]. The changes in cancer cell metabolism lead to altered production of various lipids, which serve as energy sources for rapidly proliferating tumor cells, as well as important structural and signaling components of cell membranes [3,4]. The alterations of structure and function(s) of biological membranes may, thus, have a significant impact on structures of membrane microdomains, activities of membrane-associated proteins, production of specific lipid metabolites, cell oxidative status or intracellular signaling. This may contribute to tumor heterogeneity and affect cellular responses to endogenous, as well as to exogenous stimuli, including therapeutic agents [5,6]. Recent advances in lipidomics enable complex analyses of cancer cell lipidome, which may help to identify lipid-based biomarkers and novel, lipid metabolism-related therapeutic targets [7,8,9,10].

Previous studies have indicated significant changes in phospholipid (PL) and fatty acid (FA) content, which accompany malignant transformation in tumor cells, including colon cancer [11,12]. Cancer cells use these newly synthesized FAs predominantly as building blocks for cellular membrane PLs [4]. Therefore, they often increase *de novo* FA biosynthesis, in contrast to non-malignant cells, which obtain their FAs for membrane biogenesis primarily from circulation [13]. Indeed, tumor cells often overexpress acetyl-CoA carboxylase (ACACA), ATP citrate lyase (ACLY), and fatty acid synthase (FASN), key enzymes responsible for *de novo* biosynthesis of fatty acids, which makes them attractive targets for therapeutic interventions [14]. ACLY is involved in the first step of *de novo* lipogenesis, the conversion of citrate into acetyl-CoA and oxaloacetate. Acetyl-CoA, produced by ACLY, is used as building block for long chain fatty acids and cholesterol synthesis [15]. ACACA catalyzes carboxylation of acetyl-CoA to malonyl-CoA, while FASN generates saturated fatty acids. In recent years, FA synthesis received a major attention as a potential target for cancer therapy, since it is an adaptation of tumor cells to reduced availability of serum-derived lipids within the tumor microenvironment [13]. Another important step following the synthesis of palmitic acid, is the production of monounsaturated fatty acids (MUFAs) via the activity of stearoyl-CoA desaturase (SCD), which, together with various elongases (ELOVL) and further desaturating enzymes, such as fatty acid desaturase 2 (FADS2), produce a wide spectrum of FAs in cell membranes [16]. SCD plays a major role in control of cancer cell metabolism and its inhibition has been shown to block cancer cell proliferation, reduce their survival, and limit cancer stem cells capacity to initiate tumors [17,18,19]. A number of enzymes involved in FA and cholesterol biosynthesis are regulated by the sterol regulatory-element binding proteins (SREBP), which have been found to promote colon cell proliferation and the ability to form tumor spheroids [20]. In addition to FA synthesis, the rapidly growing tumor cells can employ lipoprotein lipase (LPL), catalyzing hydrolysis of lipoprotein triacylglycerides (TAG), and fatty acid transporter CD36, in order to acquire fatty acids from blood and, thus, further fuel their growth [21]. LPL is considered to act as a metabolic gatekeeper, especially for dietary fatty acids [22]. The expression of both LPL and CD36, FA transporter, is primarily controlled by the peroxisome proliferator-activated receptor γ (PPARγ), a nuclear receptor that is frequently mutated or deregulated in colon cancer cells [23]. Overall, numerous FA regulatory proteins and FA synthesis enzymes are affected as a part of cell transformation and adaptation during cancer development [3,13].

As *de novo* lipogenesis has been shown to alter PL profiles in cancer cells [24], the deregulation of both FA synthesis and incorporation of dietary FAs into PLs can have a profound impact on colon cancer cell phospholipidome. The dysregulation of PLs in the cell membrane correlates with altered viability, proliferation and tumor development, and the biochemical changes of PL composition may take place well in advance of morphological changes observed in tumors [25]. Physical and functional interaction between membrane proteins and PLs may also affect survival, cell adhesion, and, consequently, tumor cell migration and invasion [4,26]. Previously, numerous alterations in PL metabolism have been observed in cancer cells. For example, increased levels of phosphatidylcholine (PC) and phosphatidylethanolamine (PE) have been found in colon tumors [27]. PL profiling has been proposed to discriminate tumor vs. normal tissue, as well as to help to identify specific cancer subtypes, e.g., in non-small cell lung cancer, which is characterized by decrease in phosphatidylserine (PS) and increase in phosphatidylinositol (PI), PE, and PC [28]. PL signatures *in situ* have been also successfully employed to identify invasive carcinoma and premalignant tissues in esophageal carcinoma [24]. Recently, Kitamura and colleagues have described significant differences in lysophospholipid (lysoPL) content in colon cancer [29]. Two classes of lysoPLs, which are produced by phospholipase (PLA)-mediated hydrolysis of membrane PLs, lysophosphatidylinositol (lysoPI), and lysophosphatidylserine (lysoPS) were significantly higher in colon cancer than in normal tissue [29]. Additionally, elevated levels of PC 16:0/18:1, lysoPC 16:0 and lysoPC 18:1 were detected in colorectal tumor samples [30]. This indicates that specific lysoPLs and the corresponding PLs may serve to efficiently discriminate tumor and normal colon cells; however, they play so far unknown role in colon tumor cells.

The identification of lipid species as potential tumor biomarkers may contribute to better colon cancer diagnosis, prognosis, and improvement of therapeutic strategies [31]. In our previous study, we have successfully introduced a modified methodology for isolation of EpCAM^+^ (epithelial) cells from both tumor and the adjacent non-tumor tissue of colon cancer patients [32]. Here, we used LC-MS/MS and GC/MS analyses of these purified samples to characterize PL, lysoPL, and FA profiles in normal and tumor epithelial cell populations. The detailed analyses of PLs and lysoPLs, as well as description of specific FAs indicate a significant remodeling of both FA spectrum and individual lysoPL species in colon tumor cells. Together with the characterization of genes involved in *de novo* FA synthesis, elongation and desaturation, we thus provide a complex view of FA and lysoPL/PL alterations, for the first time in purified colon cancer epithelial cells.

## 2. Results

### 2.1. Analysis of FA Profiles in Tumor and Non-Tumor Cells

**Figure 1:**
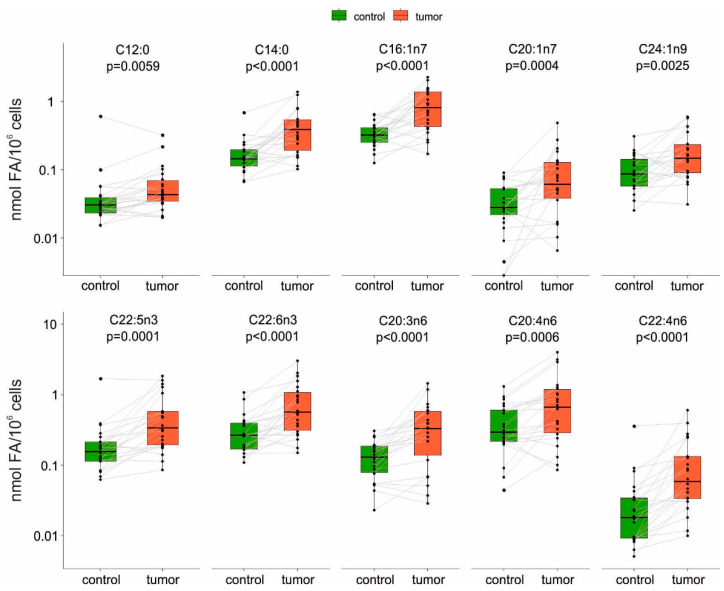
The amounts of individual fatty acids (FAs), which were found to be specifically increased in tumor EpCAM^+^ cells as compared with the cells isolated from the adjacent non-tumor (control) tissue (n = 22). Values of non-tumor and corresponding tumor cells from the same patients are linked in pairwise graphs and the respective *p*-values are indicated above each pair of box plots.

We isolated EpCAM^+^ cells from fresh tumor and adjacent non-tumor tissues (n = 22; for description of the patient cohort, see Supplementary Table S1), and we analyzed them for FA content using GC/MS. Overall, total levels of FAs were not significantly increased in isolated tumor cells. However, a significant increase was observed for the sum of n-3 polyunsaturated fatty acids (PUFA) (Supplementary Figure S1). Notably, when evaluating the amount of 40 individual FAs, significantly higher amounts of 13 individual FAs were detected, when comparing the respective tumor/non-tumor (T/N) ratios (Supplementary Figure S2). Increased levels of saturated FAs (SFA) (C12:0, C14:0, C15:0 and C19:0), MUFAs (C16:1n7, C20:1n7, C24:1n9), n-3 PUFAs (C22:5n3 and C22:6n3), and n-6 PUFAs (C18:3n6, C20:3n6, C20:4n6 and C22:4n6) were found in tumor cells. The levels of 10 prominent FAs, that were found to be significantly increased, are shown in pairwise comparison in Figure 1. Interestingly, levels of the abundant SFAs and MUFAs, such as palmitic acid (C16:0), stearic acid (C18:0), and oleic acid (C18:1) were not increased, as they were probably used as precursors for further unsaturated and very-long chain FAs. Importantly, several FAs with three or more double bonds were found to be significantly increased in isolated tumor cells, including arachidonic acid and docosahexaenoic acid. The full list of analyzed FAs, including their trivial and IUPAC names is provided as Supplementary Table S2.

### 2.2. Calculation of Enzymatic Activities Based on the Analytical Data

**Figure 2:**
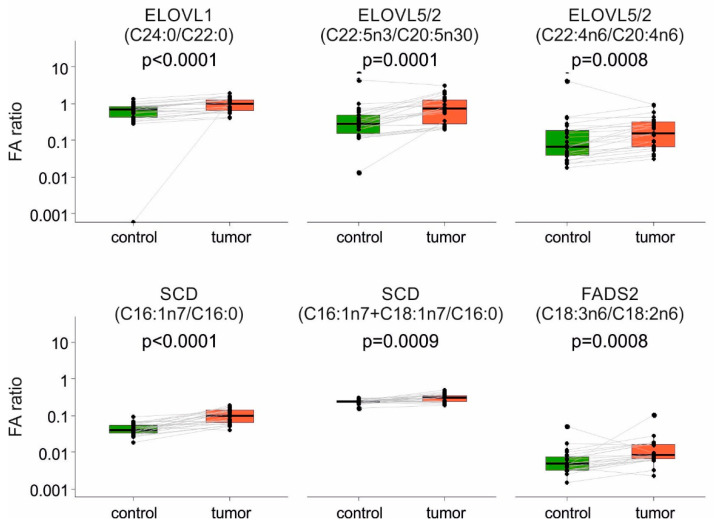
Calculated activities of enzymes involved in fatty acid (FA) synthesis, based on the amounts of individual fatty acids (product-to-precursor ratios) determined in purified non-tumor (control) and tumor EpCAM^+^ cells (n = 22). Values of non-tumor and corresponding tumor cells from the same patients are linked in pairwise graphs and the respective *p*-values are indicated above each pair of box plots. The identities of FAs used for calculations are indicated above each pair of box plots.

Determination of levels of individual FAs enabled us to calculate activities of enzymes involved in FA synthesis and metabolism, as product-to-precursor ratios. Here, significantly increased enzymatic activities (Figure 2) were found for FA elongases (ELOVL1, ELOVL5/2), as well as for FA desaturases (SCD and FADS2). Interestingly, when SCD activity was calculated based on oleic/stearic acid ratio, an opposite trend was observed, which could be due to utilization of those FAs for further enzymatic reactions. A schematic presentation of FA metabolism relevant for the results shown in Figure 1 and Figure 2, with indicated changes in FA levels, is provided as Supplementary Figure S3.

### 2.3. Expression Analysis of Genes Involved in Control of Lipid Metabolism and FA Synthesis

**Figure 3:**
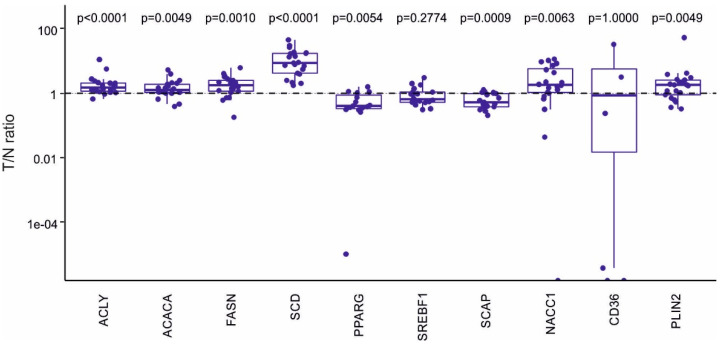
mRNA levels (tumor/non-tumor cell ratio, T/N) of the genes involved in fatty acid (FA) synthesis and control of lipid metabolism. Total RNA samples were isolated from tumor and non-tumor EpCAM^+^ cells, reverse transcribed and then analyzed using custom-made PCR arrays, using *IPO8* as a reference gene. Box plots represent data from 22 colon cancer patients. Statistical significance is indicated above each individual box plot as *p*-value.

We then analyzed mRNA levels of selected FA synthesis and lipid metabolism genes using custom-based PCR arrays. The results are displayed in Figure 3, as tumor/non-tumor (T/N) ratios for all evaluated genes.

Here, a prominent upregulation of SCD mRNA levels was observed in all tumor samples. In fact, mRNA levels of enzymes of FA synthesis pathway was significantly increased in tumor cells, including ACLY, ACACA, and FASN. In addition, NAC1 mRNA (which is encoded by *NACC1*), a transcriptional regulator of FASN in tumor cells [33], was also increased, as well as PLIN2 (perilipin 2) mRNA, which is involved in lipid droplet formation [34]. In contrast, mRNA levels of PPARγ and SCAP, which participate in the control of lipid synthesis in intestinal crypts, were both reduced in tumor cells. No significant changes were observed for SREBF1 and CD36 mRNAs.

**Figure 4:**
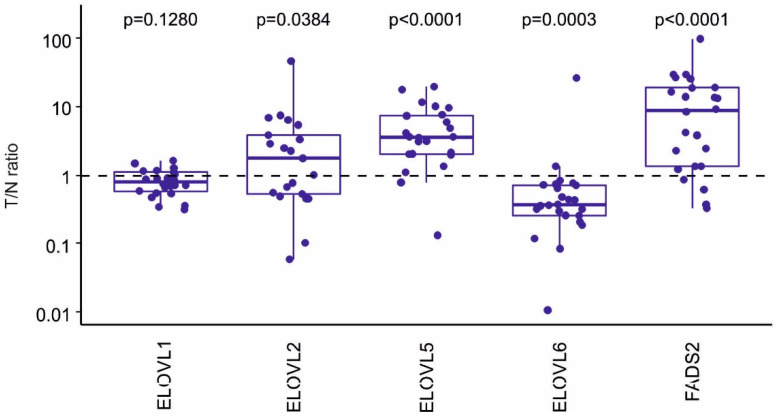
Gene expression (tumor/non-tumor cell ratio, T/N) of significantly altered mRNA levels encoded by the genes involved in fatty acid (FA) elongation/desaturation, as determined in whole tumor and respective non-tumor tissue samples. *IPO8* was used as a reference gene. Box plots represent means of 22 colon cancer patients. Statistical significance is indicated above each individual box plot.

Given the observed changes in the spectrum of FAs (Figure 1), we additionally evaluated the expression of further FA elongation and desaturation enzymes, which were not included in the original PCR array design. The results from whole tumor and non-tumor tissues are shown in Figure 4. Here, significantly increased mRNA levels of ELOVL5 were found in tumor tissue, which corresponded with increased calculated ELOVL2/5 activity. In contrast, levels of ELOVL6, which catalyzes a key step leading to the formation of stearic acid from palmitic acid, were decreased. A prominent upregulation of FADS2 mRNA was also observed, which corresponded well with its calculated enzymatic activity based on the analytical data.

### 2.4. Phospholipid Analysis of Primary Tumor and Non-Tumor EpCAM^+^ Cells

In order to determine the impact of malignant transformation on cellular phospholipidome in colon adenocarcinoma cells, we next performed analyses of PLs and lysoPLs in purified primary tumor and non-tumor EpCAM^+^ cells. The PL analyses were based on peak area values (directly derived from chromatograms) reflecting the content of individual lipid compounds (represented by specific molecular weight—*M*_W_ species), and they were normalized to an internal standard. We did not observe significant differences in total levels of any class of both PLs and lysoPLs between control non-tumor and tumor cells (Supplementary Figure S3). However, when we performed a more detailed analysis of individual PL and lysoPL species, we uncovered several significant changes. Notably, several individual PC species were significantly increased in tumor cells, including C32:0, C32:1, C36:5, and C38:4 (Supplementary Figure S4).

**Figure 5:**
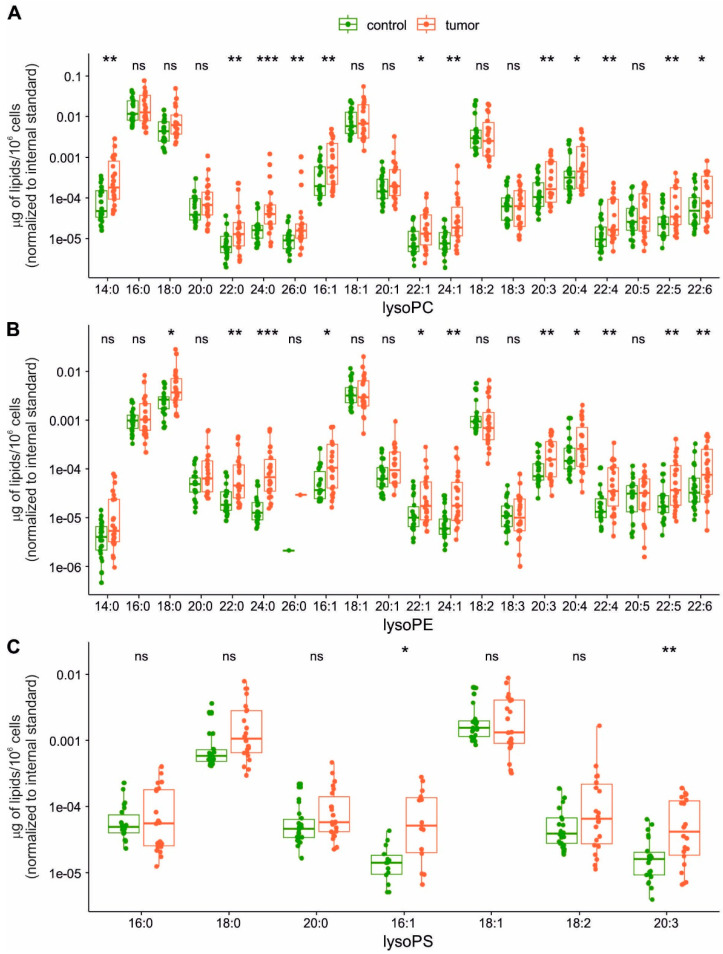
Comparison of the amount (μg of lipid/10^6^ cells) of individual lysophospholipid species (indicated as number of C:double bond number). (**A**) lysophosphatidylcholine (lysoPC), (**B**) lysophosphatidylethanolamine (lysoPE), and (**C**) lysophosphatidylserine (lysoPS) species detected in non-tumor (control) and tumor EpCAM^+^ cells (n = 22). Statistical significance is indicated above each box plot pair: * < 0.01, ** < 0.001, *** < 0.0001, ns ≥ 0.01.

We then evaluated also lysoPL/PL ratios, and here, we observed significantly higher ratio of lysoPE/PE and lysoPS/PS in tumor cells, with a similar trend (albeit not significant) also for lysoPC/PC ratios (Supplementary Figure S5). This indicated that individual lysoPL species could also be significantly altered in tumor cells. Therefore, we next performed a more detailed analysis of individual lysoPC, lysoPE and lysoPS species (Figure 5).

We found that significantly increased lysoPL species in tumor cells included particularly FAs with longer carbon chains (C20-26). These included mostly PUFAs: C20:3n6 (dihomo-γ-linolenic acid) C20:4 (n-6 arachidonic acid or n-3 eicosatetraenoic acid), C22:4n6 (adrenic acid), C22:5n3 (docosapentaenoic acid), and C22:6n3 (docosahexaenoic acid). Several lysoPLs containing very long-chain SFAs and MUFAs were also increased in tumor cells (C22:0—behenic acid, C24:0—lignoceric acid, C22:1—erucic acid, C24:1—nervonic acid). Nevertheless, increased levels were also found for C14:0 and C16:1n7 in lysoPCs, C16:1n7 and C18:0 in lysoPEs, and for C16:1n7 in lysoPE. This corresponded with an increased accumulation of palmitoleic acid (C16:1n7) in tumor cells (Figure 1).

## 3. Discussion

Colorectal cancer (CRC) remains one of major causes of cancer-related deaths globally, despite recent advances in its early screening and improved treatment options [1]. Increased numbers of CRC cases are also being diagnosed in younger patients, below 50 years of age [35]. The lipid metabolism has been proposed to have a significant impact on colon cancer development and several lipid metabolic pathways have been successfully targeted in various models of colon cancer by pharmacological inhibitors, although currently no lipid-targeting therapies are used clinically [36]. A very recent comprehensive evaluation of CRC lipidome across several independent patient cohorts has indicated that altered lipid profiles indeed represent a common feature of colon tumors [31]. Indeed, numerous types of lipids have been tested as potential CRC biomarkers, with implications for diagnostics and monitoring of the disease [6,11,30,36]. In the present study, we focused on two possible consequences of deregulated lipid metabolism in purified tumor epithelial cells: (i) deregulation of FA metabolism resulting in deregulated FA composition of tumor cells; (ii) altered lysoPL/PL ratios and increased levels of specific lysoPL species. This was based on our recent study, indicating that PL profiling can be successfully used to discriminate between normal and tumor cells isolated from CRC patients [37].

In mammalian cells, FAs are either derived from the diet or produced via *de novo* synthesis, with the latter pathway being the preferred one in cancer cells [38]. Here, FASN is a key enzyme, which catalyzes the synthesis of palmitate-CoA and palmitate is then elongated or desaturated to produce a range of very long-chain FAs. FASN uses substrates produced by the consequent activities of ACACA and ACLY enzymes, which, together with FASN have also been found to be deregulated in a number of cancer types [13]. ACLY has been previously found to be upregulated and to confer resistance to anticancer drugs in CRC cells [39]. Similar to that, inhibition of ACC has been shown to induce apoptosis in colon cancer cells [40]. Just recently, CRC tumor tissue has been found to contain highly upregulated FASN [31], which is supported by our findings both in whole tumors and purified primary tumor cells (this study; [32]). Increased expression of FASN, ACC, and ACLY, together with significantly increased levels of several types of FAs, thus confirms that *de novo* synthesis of FAs is upregulated directly in colon cancer epithelial cells, and not just in whole tumors. An increased synthesis of FAs seems to contribute partly to the control of colon tumor growth, but more importantly to the process of metastasis [41]. Nevertheless, we did not observe a general upregulation of all FAs, but rather a specific alteration of the profile of FAs. The significance of this is presently not clear. In contrast to FA synthesis genes, we found no upregulation of CD36 in the present study, which seems to support the hypothesis that colon cancer cells increase their FA content primarily via increased FA synthesis. Presently, the status of CD36 in colon cancer remains inconsistent and it may require further investigation [36]. SREBP1, which is encoded by the *SREBF1* gene, is a major regulator of FA synthesis; however, SREBF1 mRNA levels did not increase correspondingly with increased levels of FA synthesis genes. Nevertheless, expression of FA synthesis genes can also be increased via additional mechanisms, not only via SREBP1. For example, deregulation of c-Myc or mutant K-ras (frequently observed in colon tumors) can alone, or in collaboration with SREBP1, also contribute to increased lipogenesis observed in cancer cells [42,43]. Finally, estimation of SREBF1 mRNA levels may also not reflect the activity of the protein it encodes, in particular its mature cleaved form, which has been shown to play a key role in the control of metabolism in colon cancer cells [20].

Our present results also confirmed an enhanced SCD expression in purified primary colon cancer cells, as well as an increased production of numerous MUFAs and PUFAs. Together with other enzymes contributing to the observed deregulation of FA *de novo* synthesis, such as ACLY or FASN, SCD presents a potential therapeutic target. The inhibition of SCD activity or SCD knockdown has been reported to limit proliferation of various types of cancer cells, including colon cancer cells [17,44,45]. Our results seem to support the hypothesis that elevated SCD expression in colon cancer cells could be linked with increased cancer cell proliferation or survival, and adaptation to stress, resulting from increased incorporation of MUFAs, such as palmitoleic acid, into bioactive PLs, ceramides, diacylglycerols, or phosphatidic acid [46]. Along with SCD, we also found FADS2 expression and activity to be significantly increased in colon tumor cells. Our data are in line with recently observed upregulation of FASN and FADS2 in colon tumors, where particularly the overexpression of FASN was negatively associated with CRC patient survival [31]. Increased expression of FADS2 in tumors, including CRC, has been proposed to promote cancer cell proliferation *in vitro*, and tumor growth [47]. Interestingly, FADS2 is an enzyme involved in arachidonic acid production, and it may promote prostaglandin E2 production in colon tumor cells, which could be linked with increased colon cancer cell proliferation [47]. FADS2 may also contribute to production of a much wider spectrum of FAs in cancer cells as compared with their normal counterparts [48,49].

The results of our FA analysis also indicated that activities of several types of ELOVLs could be increased in colon cancer cells. A recent study has suggested that colorectal tumors exhibit significantly higher levels of 22-, 24-, and 26-carbon FAs, as well as higher levels of ELOVL1 and ELOVL6 enzymes [50]. Here, we did not observe an upregulation of either enzyme (instead, ELOVL6 expression decreased in colon tumors). However, we found a strong upregulation of ELOVL5 (corresponding with increased calculated ELOVL5/2 activity) in colon tumors. Together with FADS2 and FADS1, levels of ELOVL2 and 5 have been found to be increased in colon tumors [51]. ELOVL5 has been shown to be markedly upregulated in prostate carcinoma cells and its inhibition has been detrimental to prostate cancer cell survival [52]. The status of ELOVL6 in cancer is less clear, as both increased and decreased expression have been reported to correlate negatively with cancer progression and patient survival in breast or liver cancer [53,54,55]. The downregulation of ELOVL6 observed in our study corresponded with the lack of upregulation of stearic acid, which is a major product of this enzyme [56,57]. Nevertheless, the data on very long-chain PUFAs, as well as on activities of elongases, should be interpreted with some caution, as diet, comorbidities, and microbiota may significantly increase the variability of PUFA content in tumor tissue [31].

We found that although total levels of lysoPLs were not largely increased in purified tumor epithelial cells, the ratios of total lysoPE/PE and lysoPS/PS were significantly higher in tumor cells. This might indicate that activities of PLA enzymes catalyzing specifically lysoPE and lysoPS production might become upregulated during colon cancer development. In a previous study, total levels of lysoPS and lysoPI were found to be significantly increased in whole colon tumors, while levels of lysoPA were reduced and no changes were observed for lysoPC or lysoPE [29]. These authors have also proposed that enzymes producing lysoPL could be involved in colon cancer progression. The above data seem to point to a deregulated production of lysoPLs in colon tumor cells, which might affect their still poorly understood signaling functions [58]. Here, we observed increased levels of specific lysoPL species, especially those of lysoPC and lysoPE. The pattern of FAs present in these lysoPLs seemed to correspond with the overall alteration of FA metabolism. Of interest are increased levels of palmitoleate in lysoPLs, because this FA has been shown to be formed upon stimulation of cell growth and proliferation [59]. The increased levels of some lysoPLs may also reflect increased release of FAs (such as arachidonic acid) from membrane PLs via the activities of PLA enzymes and resulting production of bioactive eicosanoid molecules.

In conclusion, we observed a significant upregulation of genes involved in FA synthesis, elongation, and desaturation in purified primary colon epithelial cells, which corresponded with specific alterations of FA profiles. The accumulation of PUFAs included also several FAs known to be substrates used by cancer cells for generation of bioactive eicosanoids contributing to CRC development, or lysoPLs with further signaling potential. The overall trend for increased levels of longer and more unsaturated fatty acids was also evident for the FAs incorporated in lysoPL, where higher lysoPL/PL ratio was observed for many lysoPL species including very long-chain PUFAs. Future studies should address possible functional roles of the identified FAs and lysoPLs in development and progression of CRC. Nevertheless, our study, together with several other recent papers addressing alterations of lipidome in colon cancer, indicate that upregulation of FA synthesis genes and alteration of lipid profiles could be of prognostic significance and may in future help to design novel therapeutic strategies.

## 4. Materials and Methods

### 4.1. Clinical Samples

The samples were obtained from the group of 22 patients (15 males and 7 females, mean age 67.6 at the time of diagnosis) with diagnosed colon adenocarcinoma. A majority of tumors were located in sigmoideum. Among the analyzed tumors prevailed those of grade 2 and stages I, IIA, and IIIB. Moreover, the patients were characterized by other parameters including BMI, smoking status, alcohol use, comorbidities, and personal and family history, and several important biochemical parameters—for details, see Supplementary Table S1. Fresh colon tumor tissues were harvested during elective tumor removal surgery; the healthy autologous tissue removed alongside the tumor was used as non-tumor control. All human samples were obtained at the Department of Surgery of the University Hospital (Olomouc, Czech Republic), based on informed consent signed by patients. The study was conducted according to the guidelines of the Declaration of Helsinki, and approved by the Ethics Committee of the University Hospital Olomouc (protocol No. 134/14 dated 21 August 2014).

### 4.2. Isolation of EpCAM^+^ Cells from Tumors and Normal Colon Mucosa

The isolation and preparation of cells has been described previously [32]. The surgically removed tissue was cut into smaller pieces with scissors under sterile conditions and digested in HBSS medium (8 mL per 1 g of tissue; Biosera, Nuaille, France) with collagenase (2.5 mg/mL, #C2674, Sigma–Aldrich, Prague, Czech Republic) and hyaluronidase (0.5 mg/mL, #H2126, Sigma–Aldrich) at 37 °C for 2 h with shaking at 128 rpm. Homogenous mixture was incubated 10 min with DNAse (0.01 mg/mL, #11284932001, Sigma–Aldrich), mixed with PBS (1:1) and centrifuged (4 °C/1400 rpm/5 min). Supernatants were removed and pellets were re-suspended in culture medium RPMI 1640 (#51800-035, Gibco) with 20% FBS, glutamax and sodium pyruvate. Suspension was filtered through 100 μm and 70 μm filters and centrifuged (RT/1400 rpm/5 min). Erythrocytes were lysed in PBS and distilled water (1:3), mixed and again centrifuged (RT/1400 rpm/5 min). Cell pellets were then re-suspended in cold and degassed MACS buffer (MACS BSA Stock Solution, #130-091-376, Miltenyi Biotec + autoMACS Rinsing Solution, #130-091-222, Miltenyi Biotec, diluted 1:20; Miltenyi Biotec, Bergisch Gladbach, Germany). Cells were counted using CASY (model TT, Roche, Czech Republic). The 50 × 10^6^ cells were re-suspended in a mixture of 300 μL cold MACS buffer, 100 μL FcR reagent (#130-059-901, Miltenyi Biotec) for blocking receptors for non-specific binding of the antibody and 100 μL EpCAM MicroBeads (#130-061-101, Miltenyi Biotec). This mixture was incubated for 30 min at 4 °C in the dark, and then washed twice with cold MACS buffer. Re-suspended cell pellet was filtered through 70 μm filter and loaded into magnetic column (#130-042-401, Miltenyi Biotec). Epithelial cells with bound magnetic beads were captured in the magnetic column and then displaced with a piston. Isolated epithelial cells were counted, centrifuged (1400 rpm/5 min) and the pellets were stored frozen for PCR (1 × 10^6^ cells) and lipid analysis (10 × 10^6^ cells) at −80 °C.

### 4.3. Lipid Extraction and Analyses

Frozen pellets of purified EpCAM^+^ cells were warmed to RT, and appropriate volumes of internal standards were added. Methanol was added to each cell pellet in Eppendorf tube (0.5 mL) and the suspense was homogenized in sonic cold-water bath. A content of tube was then transferred into degreased glass tubes (100 × 10 mm) using a Pasteur pipette. This process was repeated three times, and the final volume of methanol suspense was 1.5 mL. After that, 0.75 mL of chloroform was added and the content was homogenized using a probe sonicator. Following the sonication, the extraction process continued at RT overnight. The solvents were then removed by drying under nitrogen, and the residue was reconstituted in 350 μL of methanol/chloroform mixture (ratio 2/1), transferred to another vial and stored at −20 °C. The number of cells for chemical analysis was limited due to the size of the tissue sample taken from the patients; therefore, only one round of lipid extraction was performed per sample. The extraction procedure was optimized to minimize a number of extraction steps, in order to prevent sample degradation. Several variants of extraction mixtures (methanol/chloroform or methanol/chloroform/water respectivelly.) have been also tested, in order to ensure a highest possible efficiency of lipid extraction. The mixture of methanol/chloroform (ratio 2/1) was selected as providing the best performance. PL and lysoPL species were then separated by a reversed-phase HPLC (Dionex Ultimate 3000 pump; Thermo Scientific) and detected by tandem mass spectrometry (MS/MS), using multiple reaction monitoring (MRM) scan mode (QTRAP 4500 with ESI; AB Sciex, Concord, ON, Canada). The following HPLC conditions were used: GEMINI column C18 250 × 4.6 mm (Phenomenex, USA), flow rate 0.7 mL/min, gradient mobile phases RA/RB (RA = methanol/water 60/40, RB = methanol, starting ratio RA/RB 60/40), total analysis time 69 min, formic acid and ammonium formate as eluent additives. The following MS/MS conditions were used: ESI in positive mode, and drying air, fragmentor voltage, and collision energy voltage were optimized for each lipid species. Mass spectrometry standards used for quantification of each assessed lipid species were purchased from Avanti Polar Lipids (Alabaster, AL, USA). Content of particular lipid species was then calculated from average chromatographic peak area and expressed as the amount of appropriate internal standard [μg/10^6^ cells] by using Analyst 1.6.2 software (AB Sciex).

Cellular FA analyses were performed using GC/MS as previously described [60]. All standards, chemicals, and solvents were obtained from Sigma–Aldrich. Sample preparation was based on a modified Bligh–Dyer extraction procedure designed for the analysis of cell pellets [61]. Cell extracts were evaporated to dryness under a stream of nitrogen, and transesterification of fatty acids was performed according to a slightly modified method of Kang and Wang [62]. Briefly, 20 μL of the internal standard solution (C23:0 fatty acid, 80 µg/mL), 20 μL butyrylated hydroxytoluene (200 μg/mL), 0.5 mL of hexane and 0.5 mL of 10% BF3 in methanol were added to the dry sample in 4 mL silanized glass vial. The vials were capped under nitrogen and heated at 100 °C for 60 min and then cooled, uncapped, and neutralized by an addition of 1 mL of a 6% solution of K_2_CO_3_. The FA methyl esters were two times extracted with 1 mL of hexane. Combined extract was evaporated to dryness under a stream of nitrogen and dissolved in 200 μL of 2,2,4 trimethylpentane. Then, 1 μL of final sample was injected to the GC/MS. GC/MS analysis was based on a combination of determination of retention times and relative abundances of selected ions. GC separation was done in an Omegawax fused silica capillary column (30 mm × 0.25 mm I.D., 0.25 μm, Supelco, Bellefonte, PA, USA). Helium at a column head pressure of 70 kPa was used as the carrier gas. An ion trap mass spectrometer Saturn 2100T (Varian, Walnut Creek, CA, USA) was used for the detection and identification of the analytes. The mass spectrometer was operated in electron ionization and scan mode at an electron energy of 70 eV.

For the calculation of enzymatic activities based on the analytical data, we used the product-to-precursor ratios of the FAs relevant for each enzymatic reaction (the particular FAs used for these calculations are shown above each box-plot in Figure 2). The selection of FAs for these calculations was based on the previously published data [63,64].

### 4.4. RNA Isolation and RT-qPCR Analysis

The relative mRNA levels of 13 genes of lipid metabolism and FA synthesis were determined by two-step RT-qPCR approach. The full list of genes used for UPL-based mRNA detection (Roche Diagnostics) is shown in Supplementary Table S3. Paired samples of patient-isolated tumor/non-tumor EpCAM positive cells were lysed and total mRNA was isolated using RNeasy Mini Kit (Qiagen, Hilden, Germany) according to the manufacturer’s instructions. mRNA was than transcribed to cDNA using Transcriptor First Strand cDNA synthesis kit (Roche Diagnostics, Mannheim, Germany) according to the manufacturer’s instructions and MJ Research PTC-200 Gradient Thermal Cycler (Bio-Rad/MJ Research, Hercules, CA, USA). The real-time qPCR was carried out with Real Time Ready Custom panels designed by Roche Diagnostic (reference number 05582610001) and LightCycler 480 Probes master (Roche Diagnostics, Mannheim, Germany) according to the manufacturer’s instructions in LightCycler 480 thermocycler. For the additional analyses of FA elongation and desaturation enzymes, archived samples of whole tumor and non-tumor tissues were used. Total RNA from deeply frozen tissue specimens was extracted with QIAshredder and RNeasy Mini Kit (Qiagen), according to the manufacturer’s instructions. The reverse transcription was performed as described above, and cDNA was analyzed using LightCycler 480 Probes master kit and LightCycler 480 thermocycler. The TaqMan™ gene expression assay (FAM) assays were provided by Thermo Fisher Scientific (Waltham, MA, USA). The list of genes, together with assay numbers, for both Roche pre-designed panels and TaqMan assays, is provided in Supplementary Table S3. In all experiments, we used IPO8 as a reference gene, based on its reported stability in various human tissue and tumor samples, including intestinal samples or colon cancer cell lines [65,66,67,68]. All changes in gene expression in tumor and non-tumor cells were calculated using the comparative threshold cycle method [69].

### 4.5. Statistical Analyses

Data from LC-MS/MS analyses were evaluated as total and subspecies levels. The total levels represent sum of all subspecies of particular lipid class. Custom PCR data and data from individual RT-qPCR gene expression assays were presented as tumor/non-tumor ratios. Statistical comparisons were done using non-parametric Wilcoxon matched-pairs signed rank test, because neither total nor subspecies levels were normally distributed. Median, interquartile range (IQR), range of non-outlying observations (i.e., values within median ± 1.5 * IQR) and original data points with linked pairwise of the same patient were shown in boxplots. Although many independent tests were performed, no multiple testing correction was applied, and, instead, *p*-values < 0.01 were considered as statistically significant. All statistical analyses were carried out using R software.

## Data Availability

The data presented in this study are available upon reasonable request from the corresponding authors.

## Supplementary Materials

The supplementary material is available online at https://www.mdpi.com/article/10.3390/ijms22136650/s1.
