# Supplementary material for: Complex Alterations of Fatty Acid Metabolism and Phospholipidome Uncovered in Isolated Colon Cancer Epithelial Cells
Source: Int J Mol Sci. 2021 Jun 22;22(13):6650. doi: 10.3390/ijms22136650 (PMC8268957; doi:10.3390/ijms22136650)
Supplement: Supplementary file 1 [file ijms-22-06650-s001.zip › ijms-1258370-supplementary.pdf]

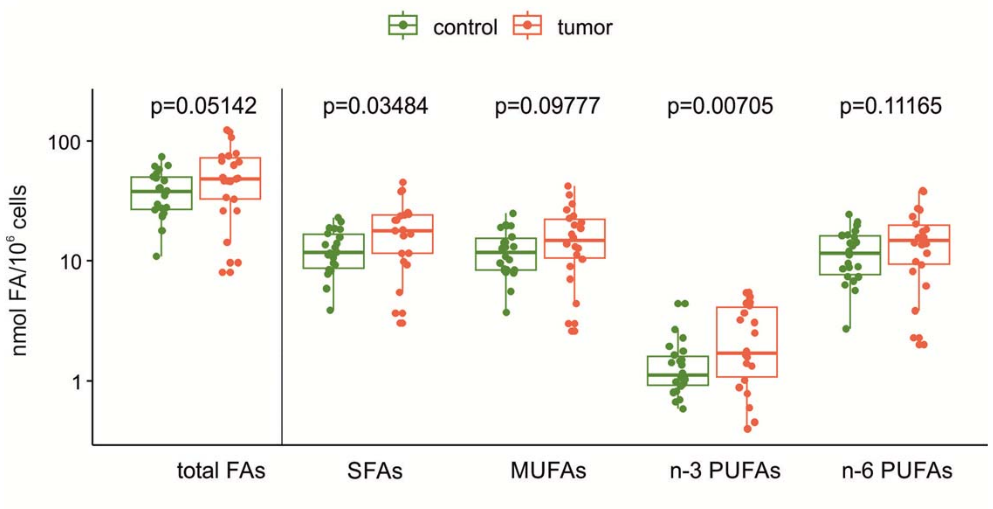

Supplementary Figure S1. Total amount of FAs and their particular classes detected in non-tumor (control) and tumor EpCAM+ cells were determined by GC/MS (n = 22). SFAs (saturated FAs), MUFAs (monounsaturated FAs), PUFAs (n-3, n-6 polyunsaturated FAs). Statistical significance (P-value) is indicated above each respective box plot pair.

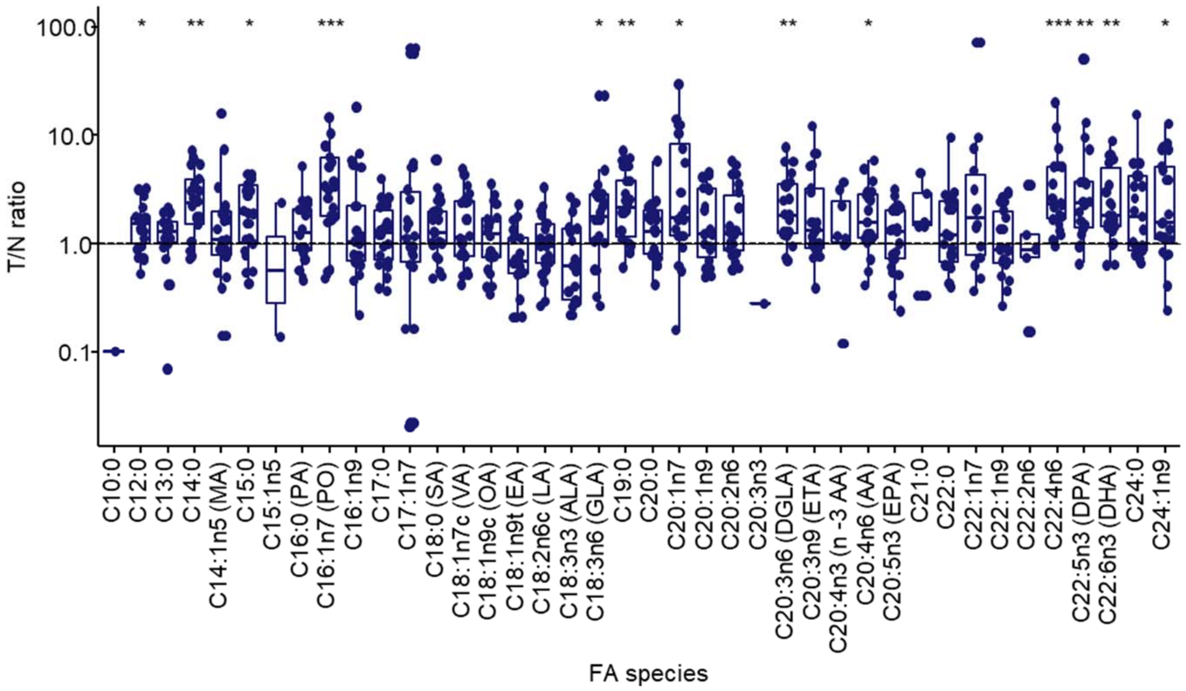

Supplementary Figure S2. Tumor/non-tumor (T/N) ratio of the amount of individual FAs detected in primary EpCAM+ cells (tumor and non-tumor) isolated from colon cancer patients. Statistical significance is indicated above each box plot:: * P<0.01 , ** P<0.001 , *** P<0.0001

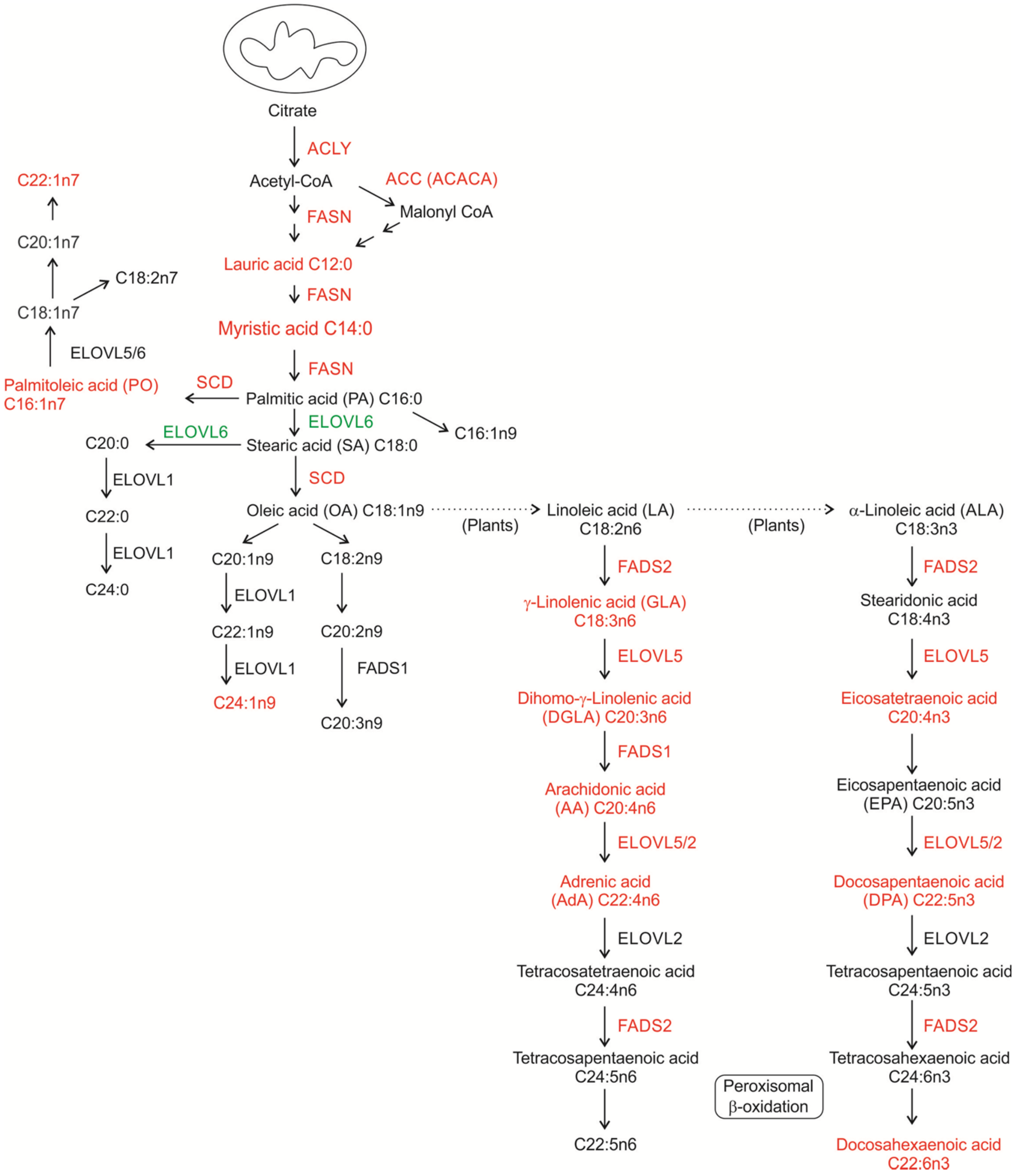

Supplementary Figure S3. A schematic presentation of FA metabolism. The scheme was prepared and modified according to Röhrig et Schultze, 2016 and Saini et Keum, 2018. Significantly up-regulated or down-regulated FAs and the genes encoding enzymes involved in FA synthesis, elongation and desaturation are indicated in red or green, respectively.

References:

Röhrig, F., Schulze, A. The multifaceted roles of fatty acid synthesis in cancer. Nat Rev Cancer 16, 732–749 (2016).

Saini, R.K., Keum, Y.-S. Omega-3 and omega-6 polyunsaturated fatty acids: Dietary sources, metabolism, and significance — A review. Life Sci. 203: 255-267 (2018).

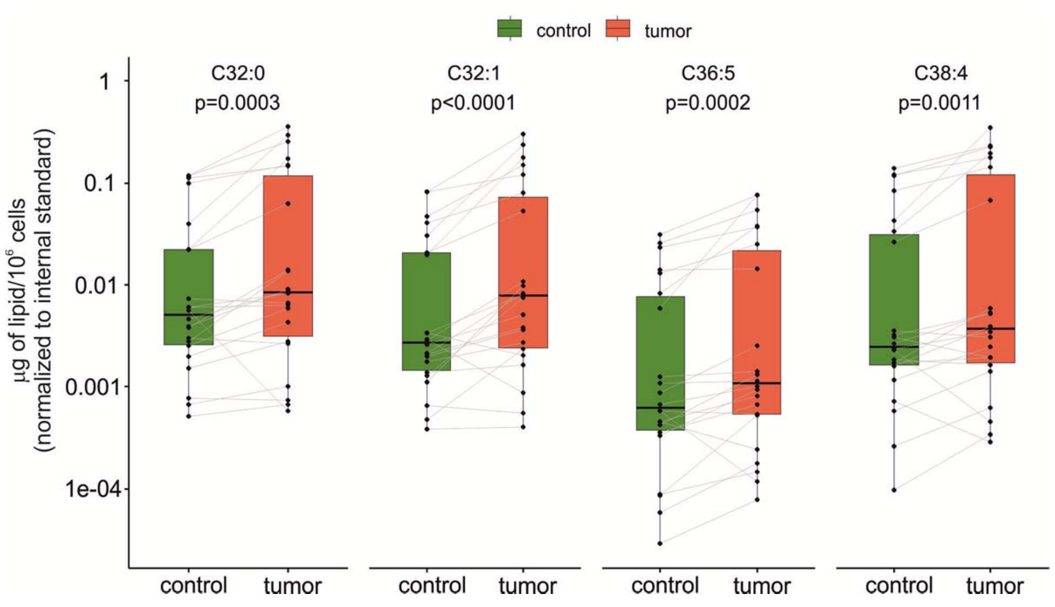

Supplementary Figure S4. PC species significantly increased in tumor cells vs. non-tumor (control) EpCAM⁺ cells, determined by LC-MS/MS (n = 22). Values of PC content in non-tumor and corresponding tumor cells from the same patients are linked in pairwise graphs and the respective P-values are indicated above each pair of box plots, together with a number of C and double bonds.

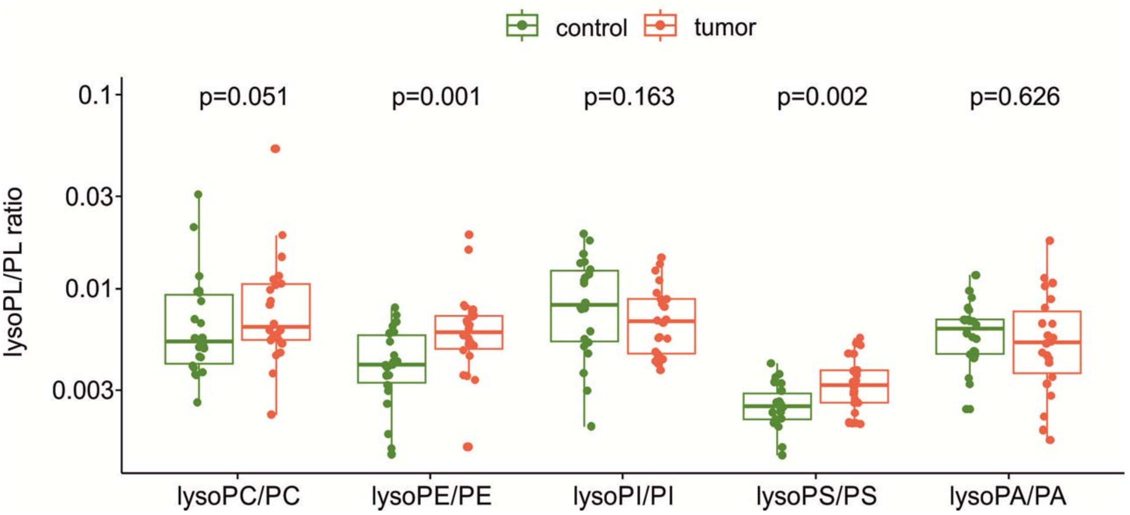

Supplementary Figure S5. Comparison of the lysoPL/PL ratio of individual classes in non-tumor (control) and tumor EpCAM+ cells (n = 22). PC, (lyso)phosphatidylcholine; PE, (lyso)phosphatidylethanolamine; PI, (lyso)phosphatidylinositol; PS,(lyso)phospatidylserine; PA, (lyso)phosphatidc acid. Statistical significance (P-values) is indicated above each pair of box plots

Supplementary Table S1. Description of patient cohort (n = 22).

Gender	
Male	15 (68.2%)
Female	7 (31.8%)
Age at diagnosis of CRC, years	67.6 (59.8 - 76.1)
BMI, kg/m2	25.1 (23 - 26.7)
<=25	11 (50%)
25-30	8 (36.4%)
>30	3 (13.6%)
Smoking status	
Current smoker	4 (18.2%)
Former smoker	2 (9.1%)
Non-smoker	16 (72.7%)
Alcohol use	
Regularly	1 (4.5%)
Occasionally	17 (77.3%)
Comorbidities	
Cardiovascular disease	11 (50%)
Respiratory disease	4 (18.2%)
Diabetes mellitus	4 (18.2%)
Personal history	
Adenomatous polyps	4 (18.2%)
CRC	0 (0%)
Non-specific intestinal inflamatory	1 (4.5%)
Other malignancy	4 (18.2%)
Family history	
Adenomatous polyps	0 (0%)
CRC	3 (13.6%)
Glucose level, mmol/L	5.3 (4.73 - 6.1)
Cholesterol level, mmol/L	4.73 (3.93 - 5.59)
HDL, mmol/L	1.09 (0.86 - 1.26)
LDL, mmol/L	2.94 (2.56 - 3.46)
TAGs, mmol/L	1.31 (1.14 - 1.56)
Albumin, g/L	36.65 (31.15 - 41.48)
Total protein, g/L	64.4 (58.27 - 71.7)
Location of colon	
Descending	4 (18.2%)
Sigmoideum	17 (77.3%)
NA's	1 (4.5%)
Grade	
G1	6 (27.3%)
G2	13 (59.1%)
G3	3 (13.6%)
Stage	
I	4 (18.2%)
IIA	8 (36.4%)
IIB	1 (4.5%)
IIIB	4 (18.2%)
IIIC	1 (4.5%)
IVA	2 (9.1%)
IVB	2 (9.1%)

Supplementary Table S2. List of analyzed FAs.

Shorthand designation	Abbreviation	Trivial Name	IUPAC Name
C12:0		Lauric acid	Dodecanoic acid
C13:0			Tridecanoic acid
C14:0		Myristic acid	Tetradecanoic acid
C14:1n5	MA	Myristoleic acid	*cis*-Tetradec-9-enoic acid
C15:0			Pentadecanoic acid
C15:1n5			*cis*-Pentadec-10-enoic acid
C16:0	PA	Palmitic acid	Hexadecanoic acid
C16:1n7	PO	Palmitoleic acid	*cis*-Hexadec-9-enoic acid
C16:1n9			*cis*-Hexadec-7-enoic acid
C17:0		Margaric acid	Heptadecanoic acid
C17:1n7			*cis*-Heptadec-10-enoic acid
C18:0	SA	Stearic acid	Octadecanoic acid
C18:1n7c	VA	Vaccenic acid	*cis*-Octadec-7-enoic acid
C18:1n9c	OA	Oleic acid	*cis*-Octadec-9-enoic acid
C18:1n9t		Elaidic acid	*trans*-Octadec-9-enoic acid
C18:2n6c	LA	Linoleic acid	*cis,cis*-Octadeca-9,12-dienoic acid
C18:3n6	GLA	γ-Linolenic acid	*all-cis*-Octadeca-6,9,12-trienoic acid
C18:3n3	ALA	*α-Linolenic acid*	*all-cis-Octadeca-9,12,15-trienoic acid*
C18:4n3	STD	Stearidonic acid	*all-cis*-Octadeca-6,9,12,15-tetraenoic acid
C19:0			Nonadecanoic acid
C20:0		Arachidic acid	Icosanoic acid
C20:1n7			*cis*-Icos-13-enoic acid
C20:1n9		Gadoleic Acid	*cis*-Icos-11-enoic acid
C20:2n6		Eicosadienoic acid	*cis,cis*-Icosa-11,14-dienoic acid
C20:3n6	DGLA	Dihomo-γ-Linolenic acid	*all-cis*-Icosa-8,11,14-trienoic acid
C20:3n3	ETE	Eicosatrienoic acid	*all-cis*-Icosa-11,14,17-trienoic acid
C20:3n9			*all-cis*-Icosa-5,8,11-trienoic acid
C20:4n6	AA	Arachidonic acid	*all-cis*-Icosa-5,8,11,14-teraenoic acid
C20:4n3	ETA	w-3 Arachidonic acid	*all-cis*-Icosa-8,11,14,17-tetraenoic acid
C20:5n3	EPA	Eicosapentaenoic acid	*all-cis*-Icosa-5,8,11,14,17-pentaenoic acid
C21:0			Heneicosanoic acid
C22:0		Behenic acid	Docosanoic acid
C22:1n7			*cis*-Docos-15-enoic acid
C22:1n9		Erucic acid	*cis*-Docos-13-enoic acid
C22:2n6		Docosadienoic acid	*cis,cis*-Docosa-13,16-dienoic acid
C22:4n6	AdA	Adrenic Acid	*all-cis*-Docosa-7,10,13,16-tetraenoic acid
C22:5n3	DPA	Docosapentaenoic acid	*all-cis*-Docosa-7,10,13,16,19-pentaenoic acid
C22:6n3	DHA	Docosahexaenoic acid	*all-cis*-Docosa-4,7,10,13,16,19-hexaenoic acid
C24:0		Lignoceric acid	Tetracosanoic acid
C24:1n9		Nervonic acid	*cis*-Tetracos-15-enoic acid

Supplementary Table S3. List of genes evaluated in individual qPCR assays.

Real Time ready - Custom panel 96-24+ (Roche, ref.No.05582610001)

Gene	NCBI Entrez Gene ID
ACLY	47
ACACA	31
FASN	2194
SCD	6319
PPARG	5468
SREBF1	6720
SCAP	22937
NACC1	112939
CD36	948
PLIN2	123
IPO8*	10526

TaqMan™ Gene Expression Assay (FAM) (Thermo Fisher Scientific, cat. No. 4331182)

Gene	NCBI Entrez Gene ID	Assay ID
ELOVL1	64834	Hs00967955_g1
ELOVL2	54898	Hs00214936_m1
ELOVL5	60481	Hs01094711_m1
ELOVL6	79071	Hs00907564_m1
FADS2	9415	Hs00927433
IPO8*	10526	Hs00914057_m1

* reference gene
